# Osteoarthritis physical activity care pathway (OA-PCP): results of a feasibility trial

**DOI:** 10.1186/s12891-020-03339-6

**Published:** 2020-05-16

**Authors:** Kelli Allen, Maihan B. Vu, Leigh F. Callahan, Rebecca J. Cleveland, Abigail L. Gilbert, Yvonne M. Golightly, Ida Griesemer, Kimberlea Grimm, Derek P. Hales, David G. Hu, Katie Huffman, Amanda E. Nelson, Ami Pathak, Jennifer Rees, Zachary D. Rethorn, Anne E. Wandishin

**Affiliations:** 1 Department of Medicine and Thurston Arthritis Research Center, University of North Carolina, 3330 Thurston Bldg, CB #7280, Chapel Hill, NC USA; 2 Center for Health Promotion and Disease Prevention, University of North Carolina, 1700 M.L.K. Jr Blvd #7426, Chapel Hill, NC USA; 3 Departments of Medicine, Orthopaedics and Social Medicine and Thurston Arthritis Research Center, University of North Carolina, 3330 Thurston Bldg, CB #7280, Chapel Hill, NC USA; 4 Department of Epidemiology, Injury Prevention Research Center, Division of Physical Therapy and Thurston Arthritis Research Center, |University of North Carolina, 3330 Thurston Bldg, CB #7280, Chapel Hill, NC USA; 5 Department of Health Behavior, Gillings School of Global Public Health, University of North Carolina, 170 Rosenau Hall, CB #7600, Chapel Hill, NC USA; 6 Department of Nutrition Gillings School of Global Public Health and Center for Health Promotion and Disease Prevention, University of North Carolina, 1700 M.L.K. Jr Blvd #7426, Chapel Hill, NC USA; 7 Medical Facilities of North Carolina, Chapel Hill, USA; 8 North Carolina Translational and Clinical Sciences Institute, School of Medicine, University of North Carolina, Brinkhous-Bullitt Building, 160 Medical Dr, Chapel Hill, NC USA; 9 Department of Orthopedic Surgery, Duke University, 311 Trent Drive, Durham, NC USA; 10 Department of Exercise and Sport Science and Thurston Arthritis Research Center, University of North Carolina, 3330 Thurston Bldg, CB #7280, Chapel Hill, NC #7280 USA

**Keywords:** Osteoarthritis, Physical activity, Pain, Function

## Abstract

**Background:**

To obtain information on feasibility and acceptability, as well as preliminary data on efficacy, of an Osteoarthritis Physical activity Care Pathway (OA-PCP).

**Methods:**

This was a single group pilot study involving 60 participants with symptomatic, physician diagnosed knee or hip OA, recruited from primary care clinics. Participants self-reported completing less than 150 min per week of moderate-to-vigorous physical activity (MVPA) at baseline. The 3-month OA-PCP intervention involved 3 physical activity (PA) coaching calls (focused on goal setting), three check-in emails and linkage with community-based or online resources to support PA. Efficacy outcomes were collected at baseline and 4-month follow-up. The primary efficacy outcome was minutes of MVPA, assessed via accelerometer. Secondary outcomes included minutes of light intensity activity, sedentary minutes, step counts, and Western Ontario and McMaster Universities (WOMAC) pain and function subscales. Participants were also asked to rate the helpfulness of the OA-PCP intervention on a scale of 0–10. Differences in efficacy outcomes between baseline and 4-month follow-up were assessed using paired t-tests.

**Results:**

Among participants beginning the study, 88% completed follow-up assessments and ≥ 90% completed each of the intervention calls. Average daily minutes of MVPA was 8.0 at baseline (standard deviation (SD) = 9.9) and 8.9 at follow-up (SD = 12.1, *p* = 0.515). There were no statistically significant changes in light intensity activity, sedentary time or step counts. The mean WOMAC pain score improved from 8.1 (SD = 3.6) at baseline to 6.2 (SD = 3.8) at follow-up (*p* < 0.001); the mean WOMAC function score improved from 26.2 (SD = 13.2) to 20.2 (SD = 12.5; p < 0.001). The mean rating of helpfulness was 7.6 (SD = 2.5).

**Conclusions:**

Results supported the feasibility and acceptability of the study, and participants reported clinically relevant improvements in pain and function. PA metrics did not improve substantially. Based on these results and participant feedback, modifications including enhanced self-monitoring are being made to increase the impact of the OA-PCP intervention on PA behavior.

**Trial registration:**

NCT03780400, December 19, 2018.

## Background

Physical activity (PA) is a core component of hip and knee osteoarthritis (OA) management, recommended across treatment guidelines [1–3]. Unfortunately, most individuals with OA are not physically active, with less than half meeting Department of Health and Human Services PA guidelines [4, 5]. There have been many trials of different PA and exercise-based interventions for knee OA, with clear evidence of improved pain, function and other outcomes when patients engage in these activities [6, 7]. However, these interventions often involve multiple in-person sessions and may not be accessible to many individuals because of program availability in their geographic area, costs, or scheduling. In addition, prior studies in this area have focused on the efficacy and effectiveness of PA and exercise programs, with little effort toward developing pathways for implementing these programs in health care systems in ways that more consistently reach and engage patients with OA [8, 9]. Because the majority of patients with OA are treated regularly in primary care, this setting provides an excellent opportunity to intervene and support these patients in increasing PA. Further, patients whose health care providers recommended PA are more likely to adhere to PA guidelines [10]. Although there is some evidence for the efficacy of primary care-based PA interventions [11–14], none of these studies have focused on patients with hip and/or knee OA, who face specific challenges such as pain, fatigue, and functional limitations. In addition, there remains a need for primary care-based PA interventions that are not only effective but also scalable and feasible for health systems to maintain in the long term.

To address these gaps, we developed and pilot tested the Osteoarthritis Physical activity Care Pathway (OA-PCP). OA-PCP was based on: 1) *Key evidence-based recommendations for PA interventions in primary care*, including: endorsement of primary providers, use of trained coaches to deliver the PA intervention, engagement of patients as active participants who select goals and identify strategies to overcome barriers, inclusion of follow-up contacts and tailored feedback, and integration with community-based and other PA resources [11, 13]; 2) *Use of a brief screening tool,* feasible to implement in primary care settings, to identify physically inactive patients who may benefit from the intervention; 3) *Inclusion of components from multiple levels of the Social Ecological Model of Health Behavior:* individual, personal, organizational and community [15, 16]; 4) *A plausible model for delivery and billing within primary care settings*: Specifically, OA-PCP was designed so that it could be delivered as part of Center for Medicare and Medicaid Services Chronic Care Management services, since OA is one qualifying health condition; 5) *Input from patients with OA and their partners, primary care providers, and representatives from community programs that provide PA resources appropriate for patients with OA.* Overall, OA-PCP was designed for widespread implementation within an existing care and reimbursement structure; it therefore has strong potential to improve PA among individuals with OA at a population health level. In this paper we describe results of a preliminary study focused on the feasibility and acceptability of OA-PCP in adults over 65 with hip and/or knee OA, as well as collection of outcome data in preparation for a larger trial.

## Methods

This study was reviewed and approved by the Institutional Review Board of the University of North Carolina at Chapel Hill. The authors followed Consolidated Standards for Reporting Trials (CONSORT) in preparing this manuscript. The study was registered on Clinicaltrials.gov (NCT03780400) on December 17, 2018, and recruitment began on March 12, 2019.

### Study design

This pilot trial was a single-group, pre-post design. We did not include a control group because the primary objectives of the study were to assess feasibility and acceptability of the intervention, rather than efficacy in comparison to a control group [17]; this was the original proposed design, approved by the funding agency. Data from this study will be used to plan a subsequent, larger trial to compare the OA-PCP intervention to a control condition. Baseline assessments were conducted following randomization, and follow-up assessments occurred after 4 months.

### Participants and recruitment

As noted above, the OA-PCP program was developed to be compatible with delivery in the context of the Center for Medicare and Medicaid Services Chronic Care Management services (https://www.cms.gov/Outreach-and-Education/Medicare-Learning-Network-MLN/MLNProducts/Downloads/ChronicCareManagement.pdf), with the rationale that chronic care managers could deliver the content of OA-PCP within broader Chronic Care Management phone calls. This would allow OA-PCP to be included within a billable service, which boosts the likelihood of downstream implementation in primary care settings. We therefore selected study eligibility criteria based on alignment with Center for Medicare and Medicaid Services Chronic Care Management service eligibility. Participants had to be age 65 or older, and have, in addition to a diagnosis of hip and/or knee OA, at least one other chronic health condition that qualified under Chronic Care Management guidelines (listed below). Other inclusion criteria were: 1) Self-reported current symptoms in a joint with OA, using the following validated item: “Do you have pain, aching or stiffness in your knees/hips on most days?” [18]. Patients also had to self-report a pain score of ≥3 on a 0–10 numeric scale (0 = no pain, 10 = extreme pain), which is an approach recommended by the Osteoarthritis Research Society International Clinical Trial Guidelines [19]. 2) Self-reported physical activity < 150 min per week, which aligns with public health recommendations. We used the Physical Activity Vital Sign (PAVS) [20–25], which includes the following two questions: 1. “On average, how many days a week do you engage in moderate to strenuous exercise (like a brisk walk)?” 2. “On average, how many minutes do you exercise at this level?” The PAVS has been implemented in a large health care system, showing good face and discriminant validity [20, 26]. We also selected this PA screening approach because it would be feasible to administer in primary care settings as part of implementing OA-PCP. Exclusion criteria for patients are listed in Table 1; these were based on safety issues related to engaging in an independent exercise program, as well as health conditions that would make it infeasible to complete other aspects of the study.

**Table 1:** Inclusion / Exclusion Criteria

**Inclusion Criteria Details**	
ICD-10 Codes for Osteoarthritis: M15.0, M16.0, M16.10, M16.11, M16.12, M16.2, M16.30, M16.31, M16.32, M16.4, M16.50, M16.51, M16.52, M16.6, M16.7, M16.9, M17.0, M17.10, M17.11, M17.12, M17.2, M17.30, M17.31, M17.32, M17.4, M17.5, M17.9, M19.90, M19.91, M19.92, M19.93	
Qualifying Comorbid Health Conditions: diabetes, depression, hypertension, hyperlipidemia, heart failure, atrial fibrillation, ischemic heart disease, stroke/transient ischemic attack, peripheral vascular disease, chronic obstructive pulmonary disease, bronchiectasis, asthma, rheumatoid arthritis, HIV/AIDS, chronic kidney disease, chronic hepatitis or osteoporosis	
**Exclusion Criteria**	
Chest pain (with physical activity or at rest)	
Loss of balance because of dizziness or loss of consciousness	
Unstable angina	
Hospitalization for cardiovascular event in last 6 months	
History of ventricular tachycardia	
Stroke with moderate to severe aphasia	
Unstable chronic obstructive pulmonary disease (2 hospitalizations within the previous 6 months and/or on oxygen)	
Dementia	
Psychosis	
Active substance abuse disorder	
Total knee or hip replacement surgery, meniscus tear, ligament tear, or other significant lower extremity injury or surgery in the last 6 months	
Planning total joint replacement in next 6 months	
Three or more falls in last 6 months	
Severe hearing or visual impairment	
Serious/terminal illness as indicated by referral to hospice or palliative care	
Recommendation from doctor to only perform physical activity under medical supervision	
Other health problem that would prohibit safe physical activity participation	
Current participation in other study related to knee or hip osteoarthritis or physical activity	
Unable to speak English	

Potential participants were first identified from among patients of participating primary care providers in 3 University of North Carolina clinics, using the electronic medical record. We first identified patients age 65 and older with diagnosis codes for knee, hip or generalized OA (Table 1), a diagnosis code for at least one qualifying comorbid health condition under Chronic Care Management guidelines (Table 1), and no diagnosis codes for exclusionary health conditions. Because we included the diagnosis code for generalized OA (to avoid missing a large number of patients with knee or hip OA), we also conducted a chart review to verify the presence of OA in a knee or hip. Primary care providers reviewed lists of patients eligible based on the electronic medical record and approved a final list of patients to contact. We mailed these patients an introductory letter, signed by their primary care provider (to illustrate providers’ endorsement and support), and then a study team member called patients to further assess eligibility. Patients who were eligible and interested in participating completed a verbal consent process and were mailed a HIPAA wavier form to sign and return. Then participants completed baseline assessments via telephone and were mailed an accelerometer for PA assessment. Participants were paid $25 for completing each phone-based assessment and $15 for returning the accelerometer at each time point. Following return of the accelerometer at baseline, participants began the OA-PCP intervention.

We aimed to enroll 60 participants in this study. As a pilot study with a focus on feasibility and acceptability, this was not powered to detect statistically significant differences in efficacy outcomes. The sample size was selected based on feasibility to complete within the study period and allowing sufficient experience to assess the feasibility and acceptability of the intervention.

### OA-PCP intervention

**Fig. 1:**
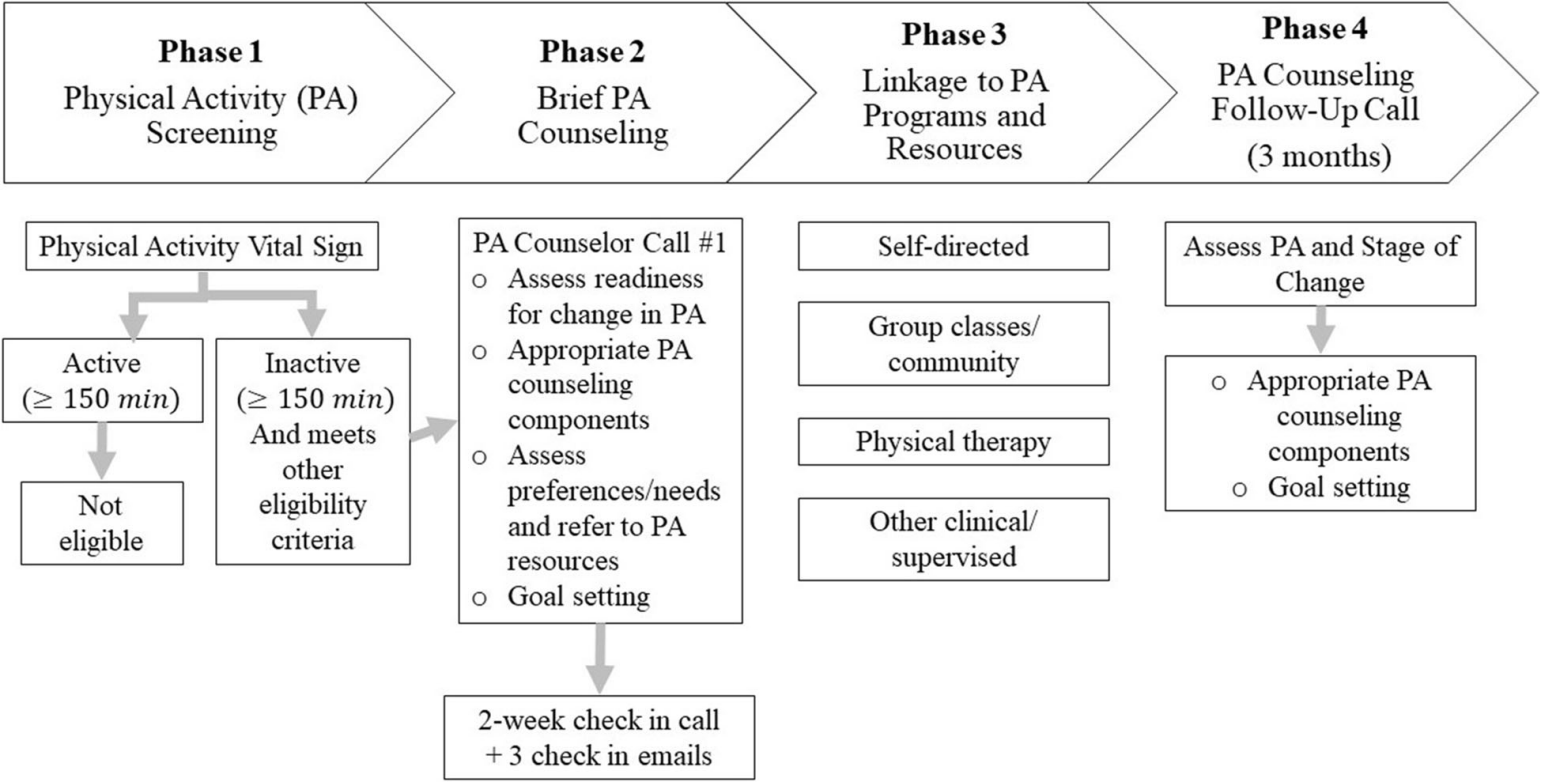
Osteoarthritis Physical Activity Care Pathway

The OA-PCP included four phases, based on previous primary care-based physical activity programs (Fig. 1) [27, 28].

*Phase 1: Physical Activity Screening*: As noted above, screening for the pilot trial was completed by the research team. However, the long-term goal is for the screening to be integrated into the primary care visit.

*Phase 2: Brief PA Coaching:* An initial PA coaching call was delivered by an individual with a health-related background who was trained in motivational interviewing concepts and the OA-PCP intervention. We aimed for this individual to have an education level similar to what is typical for Chronic Care Management counselors in primary care settings, which is often a Bachelor’s degree. The content of this initial call was designed to be brief enough that it could be embedded within a routine Chronic Care Management call. The PA coaches (one primary, one “back-up”) used detailed scripts for all intervention calls. For each call, the content was tailored according to the patient’s Stage of Change for PA, which was assessed at the beginning of the call: pre-contemplation (not ready to increase PA right now), contemplation (getting ready to increase PA, intend to within the next 6 months), preparation (ready to increase PA within the next month), action (have been increasing PA during the past 6 months) or maintenance (have been regularly active for at least 6 months, focused on avoiding setbacks) [29, 30]. The PA coaches delivered up to 4 coaching components during the first call, which were chosen based on participants’ Stage of Change [31]:

1. *Description of the Benefits and Appropriateness of PA for OA (Pre-Contemplation, Contemplation, Preparation)*: This component included a summary of benefits of PA and basic information for safe, appropriate PA in the context of OA. In accordance with OA treatment guidelines and Department of Health and Human Services recommendations [4, 32], the PA coaches recommended that participants incorporate aerobic, strengthening and stretching activities in a comprehensive approach to PA. With regard to aerobic activity, the PA coaches encouraged a long-term goal of 150 min of moderate intensity activity per week, per Department of Health and Human Services recommendations [4]. The coaches also stressed the value and health benefits of interim goals when increasing amount of weekly aerobic activity. With respect to strengthening exercises, participants were encouraged to perform these at least twice per week on non-consecutive days [4]. Participants were encouraged to perform stretching exercises daily.
2. *Discussion of Preferences for PA and Identifying Appropriate PA Resources (Contemplation, Preparation, Action, Maintenance)*: The PA coaches asked participants brief questions to understand types of PA they enjoy most and are likely to engage in. Based on this information, the coach recommended specific PA programs and resources.
3. *PA Goal-Setting (Contemplation, Preparation, Action, Maintenance)*: The PA coaches worked with participants to establish PA goals, with an initial focus on the next 2 weeks (before the next scheduled phone call; Fig. 1). The coaches also elicited potential barriers and used a problem-solving approach to address each one.
4. *Discussion of Plans for Dealing with Setbacks (Maintenance)*: For participants who were regularly active, the PA coaches gave tips for identifying setbacks early and worked with participants to identify strategies for addressing these.
  To support topics discussed during the first phone call, participants were mailed and/or emailed materials including: 1) handouts describing appropriate exercise for people with OA, tips on PA for people with arthritis (including pain management), instructions for different types of exercise (aerobic, strengthening, stretching), and example exercises; 2) a list of local and internet-based PA programs and resources (Additional file 1); and 3) a worksheet for documenting PA goals.

Approximately two weeks after the initial call, the PA coaches contacted participants to review progress toward goals, set new goals as appropriate, and check on whether they had successfully connected with PA programs or resources discussed. Participants also received a series of three check-in emails after this call, approximately 3 weeks apart. The content of the emails was tailored based on individuals’ PA goals and included: 1) a reminder of goals (if appropriate) and tips for success in fitting more PA into the day; 2) review of benefits of PA; and 3) tips for managing pain with PA.

*Phase 3: Linkage to PA Programs and Resources.*

A key aspect of this PA intervention model is connection with community-based and other resources (e.g., online programs, self-directed Walk with Ease) that can help to support patients following formal interaction with the PA coach. As described above, review of and linkage to these programs is a key component of coaching calls. The study team developed locality-specific lists of PA programs, including those specific for individuals with arthritis and more general programs and resources appropriate for this age group.

*Phase 4: PA Coaching Follow-Up.*

The PA coaches called participants approximately three months following the initial call, again using phone scripts. For participants who previously set goals and identified PA resources to engage with, the coaches assessed progress, identified additional PA resources if needed, and worked with participants to set new, longer-term PA goals. If participants did not set goals or select PA resources to try during the first two calls, they were invited to do so at this follow-up call.

### Measures

#### Feasibility and acceptability

The following metrics were specified to assess feasibility and acceptability of the OA-PCP intervention: Proportion of screened participants who are eligible and who consented to participate (along with refusal reasons for those who did not participate); Proportion of screened patients who met the PA eligibility criterion (< 150 min per week); Proportion of screened patients who were eligible overall; Proportion of participants who completed each phase of the intervention and follow-up assessments. To assess acceptability, participants were asked, “On a scale of 0-10, with 0 being not helpful at all and 10 being very helpful, how helpful was this program in supporting you to increase your physical activity?” Participants were also asked a series of open-ended questions to elicit feedback for improving the OA-PCP intervention.

### Efficacy measures

#### Primary efficacy outcome: objectively assessed physical activity

The primary efficacy outcome was minutes of moderate to vigorous intensity PA (MVPA) per week, since this corresponds to Department of Health and Human Services recommendations and is a known predictor of outcomes in patients with OA [4]. We also examined minutes of light intensity activity, sedentary minutes and step counts, as these are also important metrics of PA among individuals with OA [33, 34]. Each participant was asked to wear an Actigraph GT3X+ (Pensacola, FL) on an elastic belt, or belt clip, during waking hours for 7 days. Instructions were provided over the phone and mailed with the accelerometer in a pre-stamped / addressed return envelope. Upon return, accelerometer data were downloaded, compiled into 60 s epochs and processed to identify wear, non-wear, and sleep periods using current algorithms, logs, and visual inspection of data [35, 36]. Total and bout minutes in sedentary, light, or MVPA were estimated using cut-points developed by Troiano [35] and Matthews [37]. Outcomes were computed for participants who wore the accelerometer for 4+ days (92.9% of people had 5 + days) with 8h hours of daytime wear (86.5% of days had 10 + hours). Minute-level outcomes were standardized to a 16-h wear day to account for variations in wear time.

### Secondary efficacy outcome: self-reported pain and function

We assessed self-reported pain and physical function with the Western Ontario and McMaster Universities Osteoarthritis Index (WOMAC) subscales [38, 39], which assess pain (5 items) and difficulty with performing a range of daily activities (17 items), rated on a Likert scale of 0 (no pain or difficulty) to 4 (extreme pain or difficulty).

#### Participant characteristics

We collected the following self-reported information to characterize the study sample: age, race / ethnicity, gender, education, work status, marital status, body mass index, Self-Administered Comorbidity Questionnaire [40], number of joints with arthritis symptoms, and duration of knee / hip OA symptoms.

### Statistical analyses

Feasibility metrics (proportions) were calculated as described above. For measures of efficacy, we calculated means and standard deviations (SDs) at baseline and 4-month follow-up. We conducted paired t-tests to assess differences between pre-test and post-test MVPA and secondary outcomes. Statistical significance was assessed at the *p* = 0.05 level, though this pilot trial was not fully powered to tests for between-group differences in efficacy outcomes. For analysis of physical activity outcomes, data were removed for 1 outlier, 2 observations with improper wear (pocket), and 2 observations reporting surgery/travel during data collection.

## Results

### Participants and feasibility metrics

**Fig. 2:**
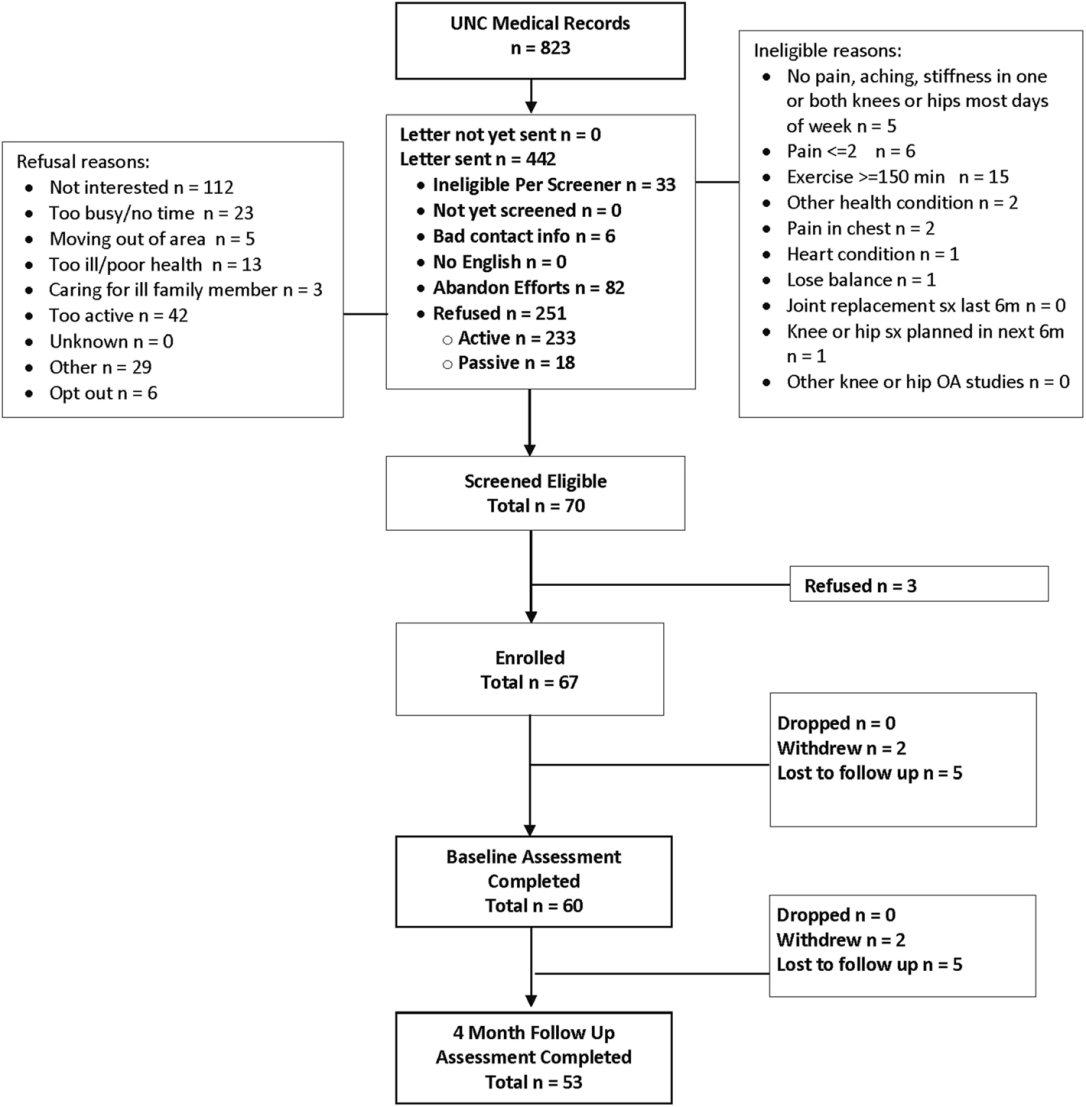
OA-PCP Study Participant Flow

We identified 823 potential participants from the electronic medical record (Fig. 2). Brief chart review determined that 381 of these potential participants failed medical record screening due to these primary reasons: no longer being active at the identified University of North Carolina primary care clinic, not having a diagnosis of knee or hip OA, joint replacement surgery scheduled during the next 6 months, knee or hip replacement surgery conducted during the past 6 months, hospitalization for stroke or heart attack in the past 6 months, on oxygen, history of falls in the past 6 months or having other health conditions that made them unable to participate in the study. The remaining 442 patients were mailed introductory letters. Of these, 251 declined participation, with the most common reasons being: not interested, self-report of being too active, too busy/no time, moving out of the area, too ill/poor health, and caring for ill family member. Among 103 individuals screened via telephone, 70 (68%) were eligible; ineligibility reasons are shown in Fig. 2. Among those eligible, 67 initially consented to participate in the study, and 7 of these could not be contacted again or withdrew before baseline assessments. Therefore, the proportion of those beginning the study (e.g., baseline assessments) out of those passing the telephone screener was 86%. However, the proportion beginning the study from among those who were mailed a letter (e.g., passed initial medical record screening) was 14%. We were particularly interested in the proportion of potential participants who meet PA screening criteria and found that among the 103 screened by telephone, only 15 reported ≥150 min of MVPA weekly. Baseline characteristics of study participants are shown in Table 2.

**Table 2:** Baseline Participant Characteristics

Mean age (SD), years	72.4 (5.6)
Gender	
N(%) Female	49 (82%)
Race	
N(%) White	41 (68%)
N(%) Black / African American	15 (25%)
N(%) Asian	1 (2%)
N(%) Other	2 (3%)
Ethnicity	
N(%) Hispanic	2 (4%)
Education	
N(%) with at least some college	48 (80%)
Marital Status	
N(%) Married or living with partner	38 (63%)
Work Status	
N(%) Working full or part time	17 (28%)
Mean (SD) Body mass index, kg/m^2^	33.4 (7.3)
Mean (SD) Number of comorbid illnesses^a^	4.3 (1.7)
Mean (SD) Years with arthritis symptoms	11.1 (9.5)
Mean (SD) Number joints with arthritis symptoms	7.7 (2.9)

^a^Possible range: 0–16. Missing data: Race: *n* = 1; Ethnicity: *n* = 9

Among those beginning the study, 53 (88%) completed 4-month follow-up assessments; 2 withdrew from the study (1 due to lack of time and one for health reasons unrelated to the study) and 5 were lost to follow-up. With respect to OA-PCP intervention delivery, 58 participants (97%) completed call #1, 57 (95%) completed call #2, and 54 (90%) completed call #3. There were no study-related adverse events.

### Acceptability measures

On the 0–10 scale regarding helpfulness of the OA-PCP program in supporting PA, the mean rating was 7.64 (SD = 2.5). When asked how the program could be improved, two of the most common participant responses were: to 1) make the program more interactive / individualized and 2) provide more prescribed PA goals. Some participants also wanted more frequent contact with the PA coach. When asked what aspects of the program helped them the most, common responses were: goal-setting, accountability, self-awareness, motivation / encouragement and information / suggestions. When asked about other information or resources the PA coach could provide, the most common answer was more individualized PA prescription. The OA-PCP intervention has been modified based on this feedback, in preparation for a larger trial.

### Efficacy measures

Table 3 shows results for primary and secondary outcome measures. At baseline, 57 participants had sufficient accelerometer wear time to be included, and at follow-up, 51 participants had sufficient wear time. Compliance with wear was exceptional. Participants averaged 7.2 days of wear (93% with 5+ days) with 13.5 h per day (87% of days had 10 + hours). The mean daily minutes of MVPA was 8.0 at baseline (SD = 9.9) and 8.9 min at 4-month follow-up (SD = 12.1, *p* = 0.515). Neither minutes of light intensity activity nor sedentary time changed considerably from baseline to follow-up, with differences < 3 min per day between time points (Table 3). The mean number of steps per day was 4392 (SD = 2217) at baseline and 4570 (SD = 2483) at follow-up (*p* = 0.368). The mean WOMAC pain score decreased from 8.1 (SD = 3.6) at baseline to 6.2 (SD = 3.8) at follow-up, indicating less pain. The mean WOMAC function score also decreased, from 26.2 (SD = 13.2) to 20.2 (SD = 12.5), indicating improved perceived physical function.

**Table 3:** Efficacy Measures at Baseline and 4-Month Follow-Up

	Baseline Mean (SD)	4-Month Follow-Up Mean (SD)	Mean Difference (Follow-Up – Baseline), 95% Confidence Interal	***p***-value
**Minutes of MVPA**	8.0 (9.9)	8.9 (12.1)	0.78 [−1.6, 3.2]	0.5145
**Minutes of Light Intensity Activity**	272.9 (81.3)	274.6 (89.7)	−0.63 [−17.3, 16.0]	0.9392
**Sedentary Minutes**	679.0 (85.1)	676.5 (93.9)	−0.15 [−17.0, 16.7]	0.9858
**Number of Steps**	4392 (2217)	4571 (2483)	143.7 [− 174.9, 462.2]	0.3683
**WOMAC Pain**	8.1 (3.6)	6.2 (3.8)	−1.9 [−2.7, − 1.0]	< 0.0001
**WOMAC Function**	26.2 (13.2)	20.2 (12.5)	−6.1 [− 8.7, − 3.4]	< 0.0001

*MVPA* Moderate to Vigorous Physical Activity

*WOMAC* Western Ontario and McMaster Universities Osteoarthritis Index; Ranges 0–20 for Pain, 0–68 for Function, higher scores indicate worse symptoms

## Discussion

This pilot study evaluated a PA intervention for patients ages 65 and over with knee and hip OA, designed for delivery within a primary care setting. Our focus in this preliminary study was on the feasibility of study processes and acceptability of the OA-PCP intervention. We were able to enroll our target number of participants within the expected time period, which supports the feasibility of a larger study of this intervention. However, 57% of potential participants who were mailed letters about the study declined to participate, and the overall proportion of participants beginning the study, among those initially identified from the electronic medical record, was 14%. We have observed similar rates of enrollment in other studies of OA interventions that have utilized the same proactive recruitment process that does not rely primarily on self-referrals [41–43]. Therefore, we do not believe the specific requirements of the study or the intervention content led to lower rates of participation compared to similar trials. Yet these recruitment metrics emphasize the importance of ensuring that study sites have a large enough patient volume to yield the targeted number of participants; information from this pilot study will inform the size of primary care clinics needed for the planned larger trial. In addition, these recruitment metrics provide some indication of the proportion of individuals who may be eligible for this intervention in both a larger trial and in a real-world clinical setting. We were specifically interested in data regarding the proportion of patients meeting the PA eligibility criterion. We found that a relatively small proportion (15%) reported ≥150 weekly minutes of MVPA, which we selected based on Department of Health and Human Services recommendations [4]. We also observed that the actual average time spent in MVPA at baseline, assessed via accelerometry, was only 8.0 (SD = 9.9) minutes daily. Based on these data, we plan to continue the screening criterion of self-reported ≥150 weekly minutes of MVPA for the larger study, rather than lower this threshold.

Metrics regarding intervention participation and follow-up assessments were very positive in this study. Proportions of participants completing each of the intervention calls were ≥ 90%; this supports the acceptability of the call length and frequency. Of participants beginning the study, 88% completed 4-month follow-up assessments. This rate is also very similar to our prior trials of behavioral interventions for patients with OA [41–43], providing support for study processes.

The mean score of overall intervention helpfulness, on a scale of 0–10, was 7.64 (SD = 2.5), which was similar to the mean rating of helpfulness in our recently completed trial of pain coping skills training for patients with OA [43]. Importantly, this pilot study allowed us the opportunity to gather actionable suggestions from participants for improving the acceptability, helpfulness, and patient-centeredness of the OA-PCP intervention in preparation for a larger study.

We did not observe any substantial changes in accelerometer-derived measures of PA following OA-PCP intervention. This was contrary to expectation and has led to changes in the intervention in preparation for a larger study. In particular, a growing body of evidence indicates that inclusion of activity trackers (e.g., Fitbits®) enhances the effectiveness of PA interventions [44–47]. Self-monitoring of PA has been identified as a critical aspect of PA behavior change [47, 48]. The OA-PCP intervention did not include any systematic process for PA self-monitoring, and we believe this contributed to the lack of change in PA metrics. In a larger study of the OA-PCP intervention, we plan to provide participants with activity trackers to facilitate self-monitoring. The goal-setting component of the OA-PCP intervention will also be modified so that participants are guided to choose goals specific to either step counts or minutes of MVPA, with long-term goals based on recent evidence for reducing risk of functional decline in OA [49, 50].

Despite the lack of change in PA metrics, participants reported clinically meaningful improvements in pain and function [51]. This could be due to the attention provided by the PA coach [52] or a Hawthorne effect [53]. The mean change in pain and function could also be a reflection of substantial changes in PA that were made by some patients.

This study has several limitations. First, as an exploratory pilot study, the trial was not sufficiently powered to detect clinically meaningful differences in efficacy outcomes. It also did not include a control group for comparing changes in efficacy outcomes. Second, participants were enrolled only from 3 clinics within one health system, which may limit generalizability. Third, the study was limited in terms of the duration of the intervention, as well as follow-up assessments. Fourth, we did not obtain radiographs from participants and therefore could not report on radiographic grade or other features.

## Conclusions

This study collected preliminary data on an intervention to enhance to PA among individuals with OA, designed for delivery in a primary care setting. Overall the pilot study supported the feasibility and acceptability of the OA-PCP intervention and study processes. The overall enrollment rate, from among patients potentially eligible for the study, was relatively low but similar to comparable studies in this area [41, 43]; this highlights the importance of engaging patient stakeholders throughout the study process to help reduce barriers to participation and maximize patient-centeredness of the intervention. Although mean self-reported pain and function scores improved, there were no meaningful changes in selected PA metrics. We believe one critical factor was the lack of a systematic process for PA self-monitoring, and this will be added via the inclusion of activity trackers in a larger study of the OA-PCP intervention. Enhancement of the OA-PCP intervention, based on results of this study, will be an important step toward building an evidence-based strategy for increasing PA and associated outcomes among the many adults with OA treated in the primary care setting. A larger trial will provide important data for health care systems regarding the effectiveness and value of implementing this program for patients with OA.

## Supplementary information

**Additional file 1.** Example list of local and internet-based PA programs and resources.

## Data Availability

The full data sets generated during the current study are not publicly available because they include personally identifiable data from study participants. However, deidentified data are available from the corresponding author on reasonable request.

## Abbreviations

MVPA: Moderate-to-Vigorous Physical Activity
PA: Physical Activity
OA: Osteoarthritis
OA-PCP: Osteoarthritis Physical activity Care Pathway
SD: Standard Deviation
WOMAC: Western Ontario and McMaster Universities Osteoarthritis Index
