# Supplementary figures and images for: Osteoarthritis physical activity care pathway (OA-PCP): results of a feasibility trial
Source: BMC Musculoskelet Disord. 2020 May 16;21:308. doi: 10.1186/s12891-020-03339-6 (PMC7229580; doi:10.1186/s12891-020-03339-6)

**Appendix A**


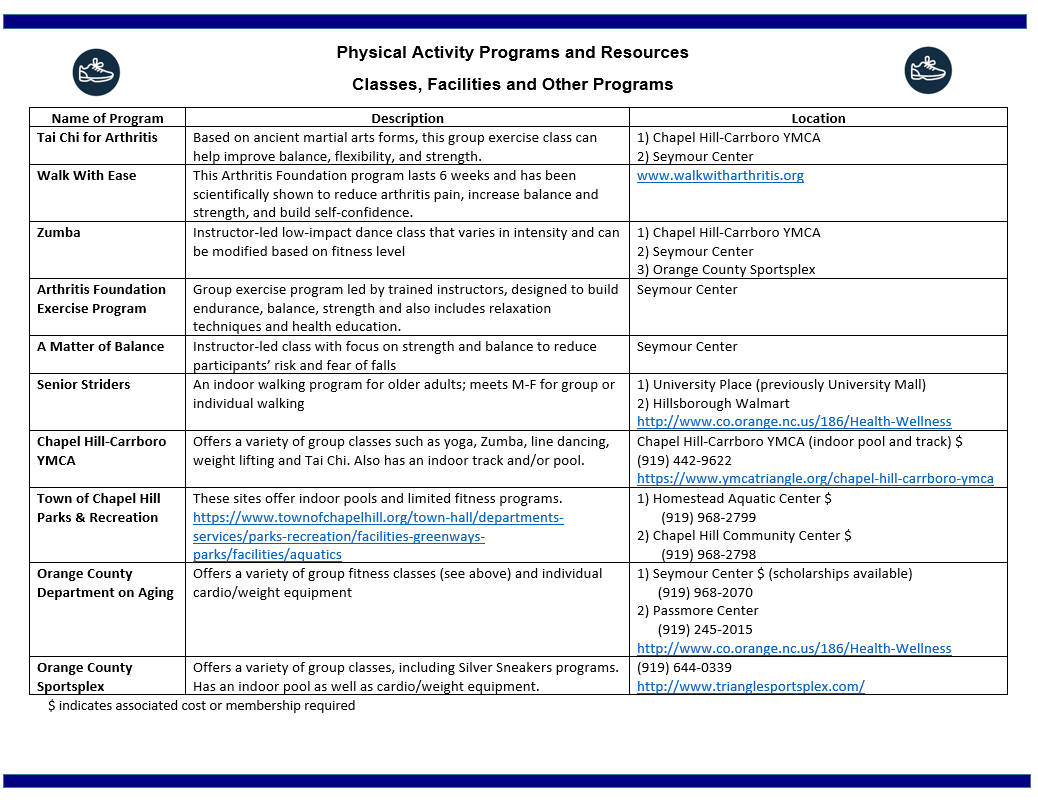

Supplement: Supplementary file 1 — Additional file 1. Example list of local and internet-based PA programs and resources. [file 12891_2020_3339_MOESM1_ESM.docx]
